# The Impact of Electromagnetic Interference from Charging All-electric Vehicles on Implantable Cardioverter-defibrillator Performance

**DOI:** 10.19102/icrm.2023.14102

**Published:** 2023-10-15

**Authors:** Abdul Wase, Umbreen Azmat Hussain, Theresa Ratajczak, Thein Tun Aung, Omair Ali, Ronald J. Markert

**Affiliations:** 1 Department of Internal Medicine, Wright State University Boonshoft School of Medicine, Wright State University, Dayton, OH, USA; 2 Department of Medicine, University Hospitals Cleveland Medical Center/Case Western Reserve University, Cleveland, OH, USA; 3 The Christ Hospital Heart & Vascular Institute, Cincinnati, OH, USA; 4 Miami Valley Hospital, Dayton, OH, USA; 5 MultiCare Medical Associates, Tacoma, WA, USA; 6 Department of Internal Medicine and Neurology, Wright State University Boonshoft School of Medicine, Dayton, OH, USA

**Keywords:** Electric vehicles, electromagnetic interference, implantable cardioverter-defibrillator

## Abstract

Electric vehicles (EVs) are growing in popularity and in general use. The effect of electromagnetic interference (EMI) caused by supercharging all-electric vehicles on implantable cardioverter-defibrillator (ICD) function has not been studied. The objective of this study was to determine the extent of the effect of EMI from charging Tesla all-electric vehicles (Tesla, Inc., Austin, TX, USA) on cardiac implantable electronic device function. A proof-of-concept study was performed to explore the potential effect of EMI from Tesla vehicles while charging the battery using a 220-V wall charger and a 480-V Supercharger. Tesla Model S and Model X vehicles were used for this study. We enrolled 34 patients with stable ICD function for the initial phase using the standard wall charger, followed by an additional 35 patients for the second phase using the Supercharger. Tracings were obtained at nominal and highest sensitivity settings while patients sat in the driver’s seat, passenger seat, back seats, and facing the charging port. In each position, the device and the patient were monitored in real time by a certified technician for any inappropriate sensing and/or delivery of therapies. A medical magnet was also available on site. Emergency medical services and physician supervision were available at all times, and patients were contacted the following day to ensure their well-being. No device interactions were identified at both the nominal and highest sensitivity settings of each ICD during exposure to vehicle charging using a Tesla 220-V wall charger and a 480-V Supercharger at any of the five positions in and around each vehicle. Interaction was defined as oversensing, undersensing, mode switch, or upper rate tracking behavior. There was also no damage to any ICD, and no inappropriate shocks were administered to any patient. In conclusion, transvenous ICD function is not interrupted by EMI transmitted while charging Tesla vehicles using either the 220-V wall charger or the 480-V Supercharger.

## Introduction

Cardiac implantable electronic devices (CIEDs) are widely used as the standard of care for the treatment of bradyarrhythmias and prevention of ventricular arrhythmias. While these devices primarily detect and interact with intracardiac electrical signals, external electromagnetic stimuli may interfere with their inherent function.

Common environmental causes of electromagnetic interference (EMI) interacting with CIEDs include cellular phones, portable headphones, security gates, electronic article surveillance devices, and headphones. Common household appliances do not cause EMI interference.^1^ In the medical field, monopolar electrosurgery and magnetic resonance imaging primarily affect permanent pacemakers. Left ventricular assist devices have reportedly impacted ventricular lead function and caused inappropriate shocks in patients with implantable cardioverter-defibrillators (ICDs).^2^

Electric vehicles (EVs) are a rapidly growing division of the automobile market and represent a movement toward environmental preservation. The Electric Vehicles Initiative, a multi-government forum launched under the Clean Energy Ministerial in 2009, involves 15 of the world’s major economies, including the United States. Sales of new electric cars worldwide in 2017 exceeded one million units—a growth of 54% since 2016—with a projected total of 220 million EVs in use globally by the year 2030 under the EV30@30 campaign.^3^

Tesla introduced its all-electric vehicle, the Model S, to U.S. consumers in 2012. Its lithium-ion battery is three to five times larger than the batteries used in hybrid vehicles. Given the concern for EMI on CIEDs, the manufacturer explicitly warns patients to stay outside of the vehicle and to avoid opening the rear hatch while charging the battery. According to the Medtronic Cardiac Rhythm Disease Management Technical Services Standard Letter (Medtronic, Minneapolis, MN, USA) released in April 2013 and the EMC Worksite Letter released in January 2009, Medtronic pacemakers/defibrillators are designed to operate normally in electromagnetic fields (EMFs) measuring 1 G (or <0.1 mT or <80 A/m) for frequencies up to 10 kHz, which includes sources transmitting 50–60 Hz alternating current (AC) power, such as motors, generators, and transformers.^4^ The Boston Scientific Cardiac Rhythm Management newsletter (Boston Scientific, Marlborough, MA, USA) from September 2013 suggested that magnetic fields stronger than 10 G can alter ICD function.^5^ The International Commission on Non-ionizing Radiation Protection 2010 Guidelines on public EMF standards specified that magnetic radiation exposure from 60-Hz sources should not exceed 2 G for the general public and 10 G for certain occupations.^6^

In 2012, Tesla introduced fast-charging Supercharger stations, of which there are now >11,000 worldwide. The effect of EMI from Superchargers on CIED function has not been evaluated. We conducted an in vivo, proof-of-concept study to explore the effects of EMI on ICD function while charging a Tesla Model S using a standard 220-V wall charger, followed by charging a Model S and a Model X with a 480-V Supercharger.

## Methods

CIEDs assessed in this study included single-chamber, dual-chamber, and biventricular ICDs from the three major device manufacturers in the United States (Medtronic, Boston Scientific, and Abbott [Chicago, IL, USA]). Exclusion criteria were abnormal baseline parameters, including abnormal lead impedances or inappropriate detection of noise on previous outpatient visits, patients younger than 18 years of age, and patients without decision-making capacity.

The primary outcome was any EMI effect on ICD function as determined by a review of device electrocardiographic tracings, such as inappropriate sensing, loss of telemetry signals, and/or inappropriate delivery or withholding of device therapies. Sweesy et al. listed the effects of EMI on ICDs^7^ **(Table 1)**.

The institutional review board of Wright State University Boonshoft School of Medicine approved the study protocols for in vivo testing. All patients signed informed consent forms prior to inclusion. Patient information was de-identified prior to the analysis.

A Trifield 100XE EMF meter (Alpha Lab Inc., Salt Lake City, UT, USA) was used to measure magnetic field strength during the charging of a Tesla Model S vehicle using the 220-V standard charger. Measurements were taken in various positions in and around the car. The highest activity of 100 mG was recorded 2 in from the charging port and near the floor of the back seat.

The study had two phases. The first phase enrolled 34 patients with stable ICD function from July 1, 2014, to June 30, 2015. A certified technician from each major manufacturer obtained the baseline parameters of each ICD. While the Tesla Model S was charged using a standard 220-V charger, ICDs were interrogated at nominal and highest sensitivity settings with patients directed to the following five different positions: the driver’s seat, front passenger seat, the right and left back seats, and directly adjacent to and facing the charging port.

**Figure 1:**
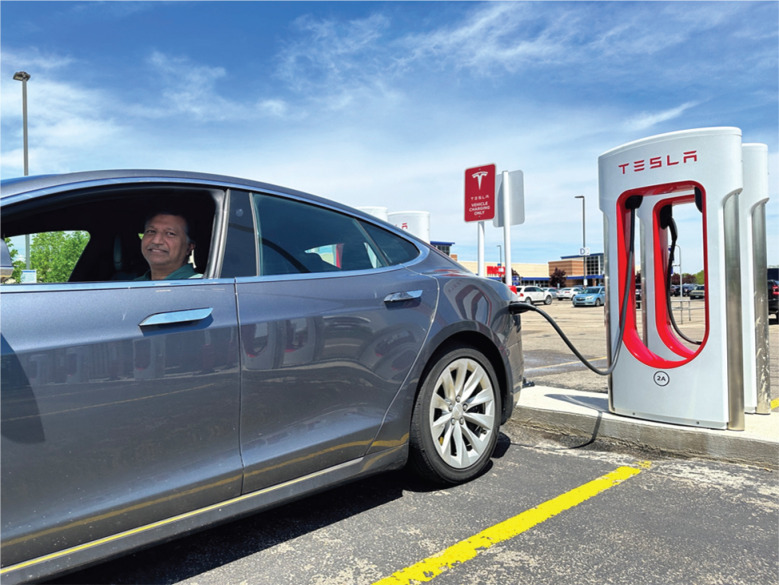
Tesla Car Model S at a Supercharger station.

The second phase occurred from July 1, 2016, to June 30, 2017 and enrolled 35 patients meeting the same aforementioned parameters. In addition to the Tesla Model S, the similarly structured Tesla Model X was used for the second testing period. ICD interrogation was performed in the same five positions. A Supercharger station delivering 480 V was used to charge the vehicle **(Figure 1)**.

Safety measures were extensively employed. Patients were monitored in real time by the available technicians for any inappropriate sensing and/or delivery of ICD therapies. A medical magnet was available at the site of testing to prevent sensing of external signals, if needed. Emergency medical services were available on site, as was a crash cart, and physicians monitored patients on site at all times. On the following day, the patient was contacted to ensure their well-being.

### Statistical analysis

The null hypothesis was that no patient would change from appropriate ICD function in the pre-treatment period to inappropriate ICD function in the post-treatment period—that is, it was anticipated that all ICDs would function appropriately in the post-intervention period.

The non-parametric McNemar test was to be used if one or more of the ICDs functioned inappropriately in the post-treatment period—ie, in statistical terms, was a failure.

Based on a clinically meaningful effect size equal to 0 (ie, no patient would experience inappropriate ICD function), we planned a sample size that would require a ≥10% failure rate for statistical significance (*P* < .05). A sample size of 30 would require four failures for a ≥10% failure rate. The eventual sample sizes were n = 34 for the first phase and n = 35 for the second phase.

## Results

In the first phase, the 34 patients had a mean age of 69 ± 9 years, 76.5% were men, and 67.6% were Caucasian. **Table 2** shows their demographic and clinical characteristics as well as ICD device information. Twenty-one percent had single-chamber devices, 32% had dual-chamber devices, and 47% had biventricular devices. Two patients (6%) were pacemaker-dependent. No patient had an identifiable device interaction at either the nominal or highest sensitivity setting during exposure to vehicle charging using a 220-V Tesla wall charger at any of the five positions tested in and around the Model S vehicle. Interaction was defined as oversensing, undersensing, mode switch, or upper-rate tracking behavior. There was also no damage to any of the ICDs, and no inappropriate shocks were administered to any patient.

The second phase of the study enrolled 35 patients, of which the majority were Caucasian (83%) and male (77%). The mean age remained 69 ± 9 years. **Table 2** shows the demographic and clinical characteristics as well as ICD device information. Fourteen percent of participants had single-chamber devices, 40% had dual-chamber devices, and 46% had biventricular devices. Fifty-one percent were pacemaker-dependent. EMI was not identified while interrogating each ICD at the nominal and highest sensitivity settings at five positions in and around both Model S and Model X Tesla vehicles, this time during exposure to vehicle charging using a Tesla 480-V Supercharger.

## Discussion

The three major types of EVs include plug-in EVs that derive power from an electric grid, EVs that are powered by electric motors and store electricity in batteries, and plug-in hybrid EVs that use batteries to power motors and use alternative fuel to power an internal combustion engine. The Society of Automotive Engineers has established standard charging power levels in the United States. Level 1 charging uses a standard 120-V household outlet and charges slowly (up to 36 h). Level 2 charging uses 208–240 V household or commercial chargers, may require dedicated equipment and connection installation, and can charge a typical EV battery overnight. Level 3 charging uses commercially available refueling points analogous to gas stations, charging a typical EV battery in <1 h at 480–600 V.^8^

Most EV batteries store energy as direct current (DC) and convert it to AC by inverter–converter units employed by the motors when driving. The Tesla Model S vehicle is equipped with a floor-mounted, 1000-lb, 400-V lithium-ion high-voltage battery that spans the length of the carriage. A charger located under the rear seat converts AC from the charging station to DC for charging the high-voltage battery. The junction box, located at the front of the vehicle, routes energy from regenerative braking, where the motor continues to spin while the vehicle is stopped, back to the high-voltage battery. Drive units located between the rear wheels and front wheels convert DC energy from the high-voltage battery into three-phase AC energy that is used to power the wheels. The Tesla Model X is similarly designed.^9^

In 2010, Ennis et al. evaluated the effects of potential EMI caused by three hybrid vehicles—2007 Toyota Prius (Toyota Motor Corp., Toyota City, Japan), 2007 Lexus RX 400h (Toyota Motor Corp.,), and 2009 Nissan Altima (Nissan Motor Co. Ltd., Yokohama, Japan)—on the function of single-chamber defibrillators that were connected to a modified pace/sense/defibrillation lead placed in various positions in the car during different driving scenarios. The highest level of EMF strength was 0.3 G while driving and 5 mG near the key holder before starting the car. There was no interference with the function of the tested devices.^10^

Tondato et al. conducted an in vivo study in 2012 to evaluate potential EMI from a 2012 Toyota Prius hybrid vehicle on ICD function. EMF was measured using a NARDA STS model EHP-50C in six positions during variable speeds of acceleration and deceleration while elevated on a four-wheel car lift, including in the driver’s seat, front passenger seat, left and right rear seats, and in front of and behind the car. The levels of EMI generated were below regulatory thresholds, and there was no evidence of oversensing or changes in ICD programming after exposure.^11^

The most recent study, published in April 2018, evaluated EMI effects on pacemaker or ICD function in 108 patients exposed to one American and three European all-electric vehicles (Tesla Model 85S, BMW i3 [BMW, Munich, Germany], Nissan Leaf, and Volkswagen e-up [Volkswagen Group, Wolfsburg, Germany]). EMF strength was measured in and around the cars during test bench operation, along charging cables, and inside cars during open-road driving. There was no evidence of oversensing or undersensing, inappropriate pacing or pacing inhibition, or device reprogramming caused by EMI from any of the vehicles.^12^

We found no EMI interference with ICD function from charging Tesla vehicles at 220 or 480 V. While this study is limited by its moderate sample size, the results suggest that patients with ICDs can safely charge all-electric vehicles despite manufacturer recommendations stating otherwise. Our findings are consistent with those of other studies conducted with hybrid and all-electric vehicles.^10–12^ Finally, additional studies are needed to further evaluate the effects of EMI on CIEDs, including pacemakers, from EV charging, especially with Supercharger stations.

## Conclusions

In a proof-of-concept study, we found that ICD function was not affected by EMI while charging Tesla vehicles using 220- and 480-V charging stations.

## Figures and Tables

**Table 1:** Effects of Electromagnetic Interference on Implantable Cardioverter-defibrillators^7^

Asynchronous pacing
Inhibition of appropriate pacing
Ventricular pacing at upper tracking rate
Inhibition or deactivation of tachyarrhythmia therapy
Inappropriate shocks
Permanent damage to the CIED

*Abbreviation:* CIED, cardiac implantable electronic device.

**Table 2:** Demographic and Clinical Characteristics for Subjects in Two Electromagnetic Interference/Electric Vehicle Test Phases

	Phase 1: 220 V Wall Charger	Phase 2: 480 V Supercharger
Age, years, mean ± SD (range)	69 ± 9 (48–85)	69 ± 9 (46–86)
Male sex, n (%)	26 (76.5)	27 (77.1)
Ethnicity, n (%)		
Caucasian	23 (67.6)	29 (82.8)
African American	10 (29.4)	5 (14.3)
Asian	1 (3.0)	1 (2.9)
Device type, n (%)		
Single-chamber ICD)	7 (20.5)	5 (14.3)
Dual-chamber ICD	11 (32.3)	14 (40.0)
Biventricular ICD	16 (47.1)	16 (45.7)
Device manufacturer, n (%)		
Medtronic	13 (38.2)	11 (31.4)
Boston Scientific	15 (44.1)	18 (51.4)
Abbott	6 (17.6)	6 (17.1)
Indication for ICD, n (%)		
Primary prevention	10 (29.4)	21 (60.0)
Prior tachyarrhythmia (secondary prevention)	15 (44.1)	12 (34.3)
Syncope	9 (26.4)	2 (5.7)
Underlying rhythm, n (%)		
Sinus rhythm	32 (94.1)	18 (51.4)
Paced rhythm	2 (5.9)	17 (48.6)

*Abbreviations:* EMI, electromagnetic interference; EV, electric vehicle; ICD, implantable cardioverter-defibrillator; SD, standard deviation.
